# Evaluation of tumor volume reduction of nasal carcinomas versus sarcomas in dogs treated with definitive fractionated megavoltage radiation: 15 cases (2010–2016)

**DOI:** 10.1186/s13104-018-3190-3

**Published:** 2018-01-24

**Authors:** Matthew J. Morgan, David M. Lurie, Armando J. Villamil

**Affiliations:** 1 Department of Surgery Affiliated Veterinary Specialists, 9905 S US Hwy 17-92, Maitland, FL 32751 USA; 2 Department of Oncology Affiliated Veterinary Specialists, 9905 S US Hwy 17-92, Maitland, FL 32751 USA

**Keywords:** Nasal carcinoma, Nasal sarcoma, Radiotherapy, Volume

## Abstract

**Objective:**

Local control is a major challenge in treating canine nasal tumors, and cytoreduction following radiation therapy has been recommended to extend survival and to delay local recurrence. Our objective was to compare the effect of definitive radiotherapy on the tumor volume of intranasal carcinomas compared to sarcomas. We evaluated 15 dogs that received radiotherapy within 1 month of initial CT scan, and post radiation CT scans performed within 3 months of completing full course definitive megavoltage radiation. Tumor reduction volume based on CT scans were obtained and compared between carcinoma and sarcoma groups.

**Results:**

The following tumor types were treated; carcinoma (8/15), sarcoma (7/15). The mean nasal tumor size before radiation therapy was 24.5 cm^3^ and tumor size after radiation therapy was 13.5 cm^3^ resulting in a mean reduction of 55.1% reduction in tumor size for both carcinomas and sarcomas. The carcinoma group displayed a volume reduction of 67.1% (SD ± 16.9) and the sarcoma group displayed a volume reduction of 21.3% (SD ± 39.7). Within the study period carcinomas were more responsive in the reduction of volume than sarcomas with fractionated megavoltage radiation.

**Electronic supplementary material:**

The online version of this article (10.1186/s13104-018-3190-3) contains supplementary material, which is available to authorized users.

## Introduction

Primary intranasal tumors account for 1% of all neoplasms that occur in the dog [1]. Due to a high recurrence rate, which exceeds 60%, treatment recommendations are focused on local disease control [2, 3]. Carcinomas represent nearly two-thirds of all intranasal tumors with sarcomas compromising the majority of the remaining tumors [3, 4]. Carcinomas and sarcomas in the nasal cavity are characterized by progressive local invasion and a typically low metastatic rate [5].

Treatment recommendations for intranasal tumors have evolved over the past 40 years with developing technology [5]. Today, the current standard of care includes radiotherapy as the treatment of choice with select authors recommending cytoreduction of the nasal cavity following radiation therapy if residual tumor volume is present [6–17].

The 2005 retrospective study by Adams et al. recommended patients pursue surgical treatment of residual intranasal disease if tumor volume regression was judged to be less than 80% based on computed tomography (CT) or if recurrence or progression of the tumor was suspected [10]. If greater than 80% tumor volume reduction was noted then a follow up computed tomogram was recommended in 6 weeks to assess volume.

To the authors knowledge, there has not been a study that compares the tumor volume reduction of intranasal carcinomas and sarcomas following definitive radiation therapy based on volumetric analysis using CT. We suspect carcinomas will have a greater decrease in volume to radiation based previously reported tumor behavior elsewhere in the body [11]. The purpose of this study was to compare the effect of definitive megavoltage radiotherapy on the tumor volume of intranasal carcinomas compared to sarcomas using readily available volumetric evaluation software. The authors hypothesized that carcinomas would have greater volume reduction based on CT volumetric analysis than sarcomas.

## Main text

### Methods

Medical records of 23 dogs with intranasal tumors that were treated with definitive radiation therapy were reviewed. Patients were eligible for inclusion if they had a histopathological diagnosis of nasal carcinoma or sarcoma, received definitive fractionated radiation treatment, and had pre and post radiation CT scans between January 2010 and July 2016. During the time period studied all clients with patients receiving radiation therapy for intranasal tumors were advised to have a post radiation therapy CT scan performed 6 weeks after the completion of treatment regardless of pre or post radiotherapy clinical signs. A pre CT scan was performed within 2 weeks of the initiation of radiotherapy. Post CT scans were completed within 1–3 months from completion of radiotherapy. Patients with a CT scan performed after the 3 month deadline were excluded from this study. Patients under treatment for other concurrent neoplasia were excluded from the study. Median survival time was defined as the number of days from the start of radiation therapy to death. Patients were staged at the time of initial CT scan and post radiotherapy scan using a previously described modified Adams staging system with stages I–IV [2]. At the time of follow up CT imaging, the clients were given the option of surgery (dorsal rhinotomy) under the same anesthetic event for cytoreduction of the remaining tumor volume.

All radiation treatment planning was done using a radiation treatment planning system (TiGRT radiotherapy treatment planning system, LinaTech Inc., Sunnyvale, CA). All 3D conformal radiation therapy (3DCRT) plans were calculated with 6 MV photons and consisted of 2 fields with the exception of two patients with 3 fields (dorsal, left and right lateral). Treatment was performed isocentrically with 1–0.5 cm bolus and wedges to ensure dose homogeneity. Patients were administered between 16–18 treatments with a median of 18 treatments. All radiation treatments were divided in 3 Gy fractions and performed once daily, Monday through Friday. The patient’s mean total cumulative dose was 52.5 Gy (range 48–54 Gy) with a median of 54 Gy. Patients with complete medical records (14/15) had a mean minimum planning target volume (PTV) of 46.6 Gy (range 39.5–52 Gy) with a median of 48.3 Gy. The mean maximum PTV was 58.4 Gy (range of 53.7–63.5 Gy) and median of 58 Gy. The mean PTV_50_ was 55.1 Gy (range of 51.3–58.1 Gy) and median was 55.7 Gy. No statistical differences were noted in the cumulative Gy or PTV_50_ between the carcinoma and sarcoma group. The mean duration of radiation therapy was 24 days (range of 20–30 days) for the study. The mean duration of radiation therapy was 25 days (range of 20–30 days) for the carcinoma group and 23 days (range of 20–26 days) for the sarcoma group.

Initial histopathology of each nasal tumor was provided by either rhinoscopy or CT guided nasal biopsy utilizing cup biopsy forceps. Confirmation histopathology was obtained after radiotherapy via dorsal rhinotomy for cytoreduction on all patients after radiotherapy.

**Fig. 1:**
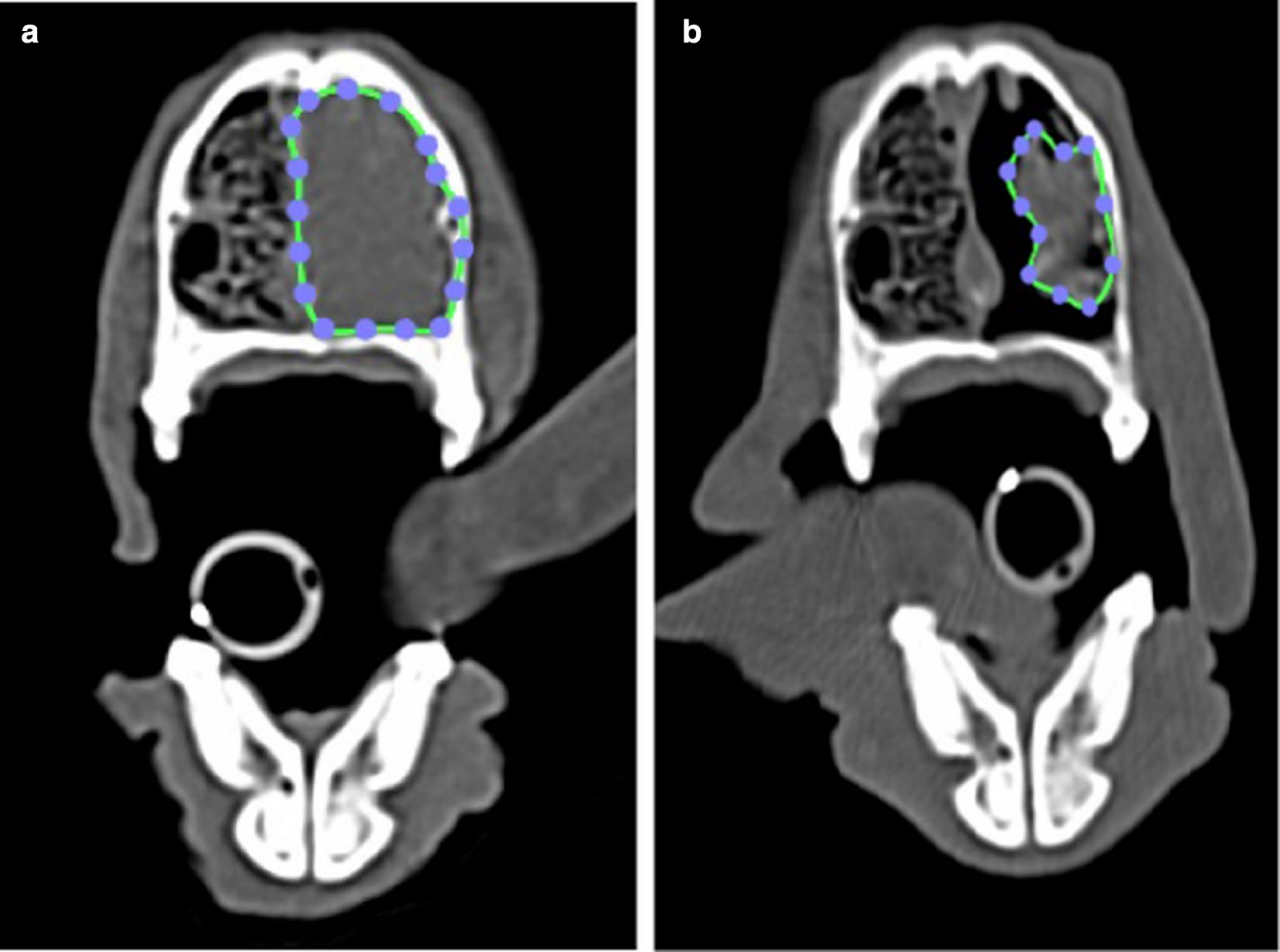
(Patient #1) **a** pre radiotherapy CT scan, **b** post radiotherapy CT scan (1 month post radiation therapy)

**Fig. 2:**
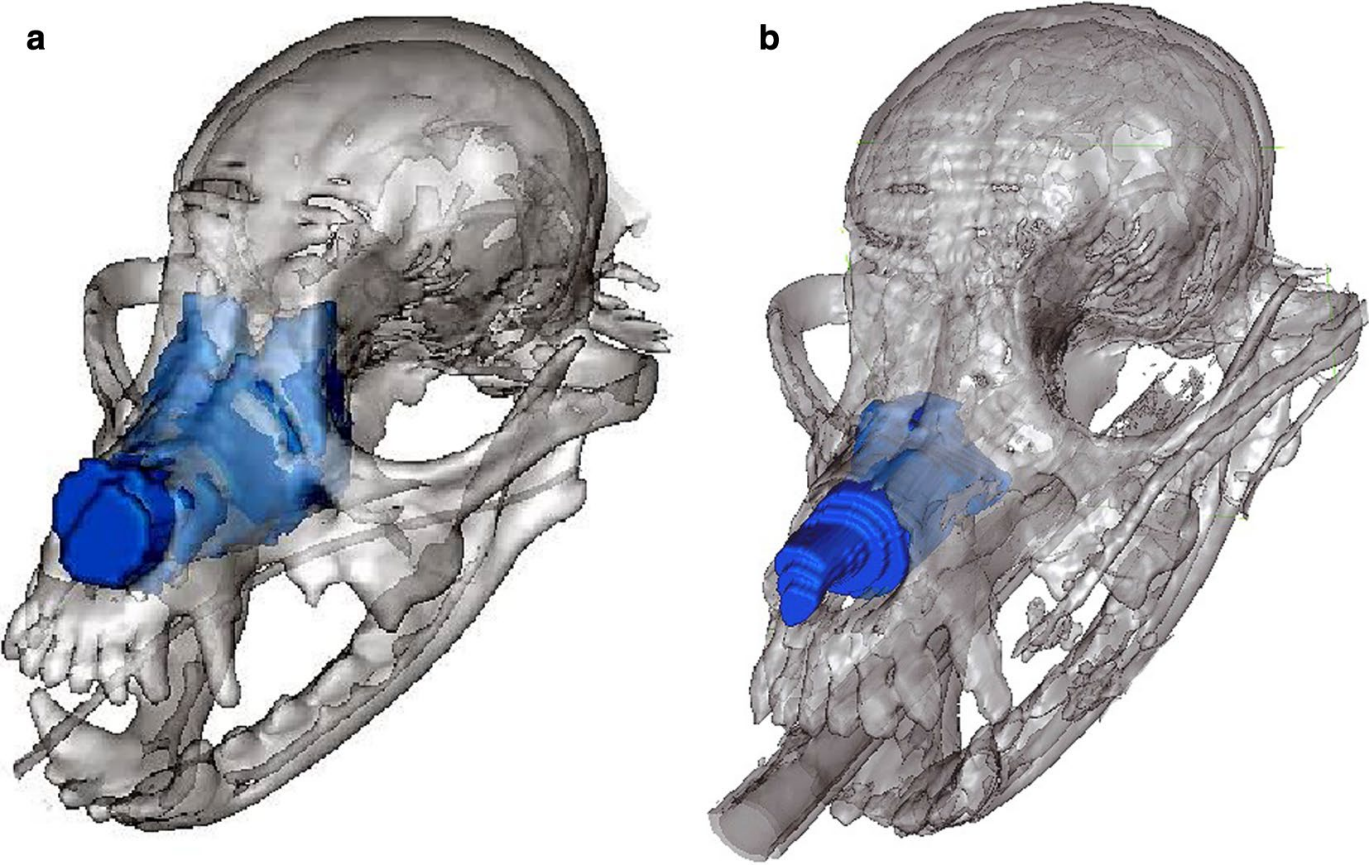
(Patient #7) **a** pre radiotherapy CT scan, **b** post radiotherapy CT scan (1 month post radiation therapy)

Volume measurements were performed by a single author (MM) using an open source image analysis software package Osirix PAC version 8.0.2. Measurements of the nasal tumors were taken using a closed polygon region of interest that was measured from each 2 mm CT slice through the entire nasal cavity using the pre and post radiotherapy CT scans as seen in Fig. 1. CT scans with 2 mm slice thickness were all performed with the same scanner (Brilliance 16 CT, Koninklijke Philips, Amsterdam, Netherlands). Nasal fluid and nasal discharge was not included in the volume measurements. An intravenous contrast agent as well as Hounsfield units were used to discern solid tumor from fluid. Tumor volume and entire nasal cavity volume of the tumor was generated from each CT study as seen in Fig. 2.

Statistical analysis was carried out using the t test for volume significance, the Kaplan–Meier survival analysis, Shapiro–Wilk Normality Test, Equal Variance Test, and Log-Rank Test for survival comparison (Additional file 1: Figure S1). Values of P < 0.05 were considered significant.

### Results

Fifteen patients met the inclusion criteria for this study. Eight were male neutered and seven were female spayed. Average age of the patient at the time of diagnosis was 10 years old (range 5–15 years) as seen in Table 1. Twelve breeds were represented including: three Beagles (n = 3, 20%), two Jack Russell Terriers (n = 2, 13%), and two mixed breed dogs (n = 2, 13%). The following tumor types were treated; carcinoma (n = 8), sarcoma (n = 7).

**Table 1:** Patient Information collected from retrospective review

Patient	Breed	Age (years)/sex	Pre RT stage	Post RT stage	Pre RT histopathology	Post RT histopathology	Volume pre (cm^3^)	Volume post (cm^3^)	Volume change %	Total nasal volume (cm^3^)	MST (days)
1	Springer Spaniel	15 FS	I	I	Carcinoma	Carcinoma	27.2	5.3	− 80.10	66.9	487
2	Collie	13 FS	I	I	Carcinoma	Chondrosarcoma	11.1	4.9	− 55.80	83.4	467
3	Labrador Retriever	10 FS	I	I	Carcinoma	Carcinoma	36.5	6.3	− 82.70	118.6	406
4	Border Collie	13 MN	I	I	Chronic rhinitis	Adenocarcinoma	31.9	9.9	− 69.00	93.3	1141
5	Jack Russell Terrier	9 MN	III	III	Carcinoma	Carcinoma	21.9	4.2	− 80.80	48.4	266
6	Beagle	14 MN	II	II	Carcinoma	Carcinoma	17.4	6.8	− 60.90	57.7	435
7	Poodle	10 MN	II	III	Carcinoma	Carcinoma	11.1	6.7	− 39.60	14.7	324
8	Wheaton	12 MN	III	II	Myxosarcoma	Fibromyxosarcoma	30.9	5.7	− 81.60	92.5	435
9	Mixed breed	8 MN	II	II	Carcinoma	Carcinoma	13.5	3	− 77.80	62.3	Alive
10	Papillon	6 MN	II	I	Periglandular fibrosis	Chondrosarcoma	10.9	9	− 17.40	26.2	Alive
11	Mixed breed	9 FS	II	II	Osteosarcoma	Osteosarcoma	50.9	36.2	− 28.90	125.2	286
12	Jack Russell Terrier	14 FS	II	II	Chondrosarcoma	Chondrosarcoma	15.4	15.5	0.60	30.0	240
13	Beagle	11 MN	II	II	Carcinoma	Adenocarcinoma	10.4	5.7	− 45.20	42.2	Alive
14	Pitbull	6 FS	I	I	Sarcoma	Sarcoma	60	56.5	− 5.80	113.1	Alive
15	Boxer	5 FS	II	III	Chondrosarcoma	Chondrosarcoma	19.2	26.3	40.00	50.4	Alive

Staging using Modified Adams staging system (I–IV), MN, male neutered; FS, female spayed; MST, median survival time, RT, radiotherapy

The mean nasal tumor size before radiation therapy was 24.5 cm^3^ (range 10.4–60 cm^3^) and tumor size after radiation therapy was 13.5 cm^3^ (range 14–113.1 cm^3^) resulting in a mean reduction in tumor size of 55.1% in both carcinomas and sarcomas. The carcinoma group displayed a volume reduction of 67.1% (SD ± 16.9 cm^3^) and the sarcoma group displayed a volume reduction of 21.3% (SD ± 39.7 cm^3^). The two-tailed P value for the volume reduction in carcinomas compared to sarcomas equals 0.010 which indicated the difference in mean values of the two groups is greater than would be expected by chance. There was a statistically significant difference between groups at a P value < 0.05. The data passed the Shapiro–Wilk Normality Test as well as the Equal Variance Test. The data repeatability was evaluated with the intraclass correlation coefficient (ICC) which displayed excellent intra-rater agreement.

The mean tumor size relative to the total nasal cavity volume was 37.8% prior to radiotherapy. Following radiotherapy the mean tumor size relative to the total nasal cavity volume was 22.3%. The smallest tumor relative to total nasal cavity size prior to radiotherapy was 13% in patient #2 and the largest was 75% in patient #7. Following radiotherapy the smallest tumor relative to total nasal cavity size was 5% in patient #9 and the largest was 51% in patient #15. There were no statistically significant differences in survival time relative to volume reduction or tumor size relative to the nasal cavity.

The mean time from pre CT scan to post CT scan was 63 days (range of 40–115 days) for both groups. The mean time from CT scan to post CT scan was 63 days (range 50–120 days) for carcinomas and 59 days (range 40–115 days) for sarcomas which was not statistically significant (P = 0.288).

All patients in the study group had surgical cytoreduction of the nasal cavity via dorsal rhinotomy to remove all visible tumor tissue. All clients elected to have surgical cytoreduction following post CT imaging during the same anesthetic event. All surgical procedures were performed by a board certified surgeon or surgery resident. The median time from completion of radiotherapy to surgery was 40 days for both groups (43 days for the carcinoma group, 37 days for the sarcoma group). There was no statistical difference from the end of radiotherapy to surgery between the carcinoma and sarcoma groups based on the Log-Rank Test.

At the time of the data collection 10 patients were deceased and 5 were still alive. The median survival time was 435 days for both groups. The median survival time for the carcinoma group (n = 6) was 435 days. The median survival time for the sarcoma group (n = 4) was also 435 days. There was no difference in overall median survival between carcinoma and sarcoma with a P value of 0.790.

### Discussion

This study was intended to compare the volumetric response to radiation therapy comparing carcinomas (67.1% reduction) to sarcomas (21.7% reduction). The results indicate a significant statistical difference in the volume reduction of carcinomas compared with sarcomas in the time frame studied. Due to the fact that tumor recurrence is the leading cause of treatment failure the recommendation of surgical cytoreduction is a logical option to slow tumor re-growth [10, 12]. Two patients diagnosed with nasal sarcoma displayed tumor growth despite radiation therapy, suggesting not all nasal sarcomas are radiation sensitive at the doses used and the time frame studied.

The cells remaining after any dose of radiation are termed the surviving fraction [18]. Tumor sensitivity to radiation has been described previously as the α/β ratio. Tumors with a high α/β ratio correspond to cell death in a linear fashion, while tumors with a low α/β ratio have a quadratic component and cell death increases in proportion to the square of the dose [18]. Previous studies suggest some sarcomas including soft tissue sarcomas and osteosarcomas, have a low α/β ratio [19]. A greater α/β ratio in carcinomas compared to sarcomas could explain why we observed greater volume reduction in carcinomas. Late responding tumors with a low α/β ratio demonstrate increased survival at low radiation doses and significantly greater toxicity at higher doses [19]. In a study comparing volume response to radiotherapy in canine brain tumors greater volume reduction was also seen in patients with carcinomas versus patients with sarcomas [11].

Based on these results the authors suggest that carcinomas are more radiation responsive than sarcomas with respect to the reduction of tumor volume observed in the initial 6 weeks following therapy. Patients diagnosed with nasal sarcomas in this study have on average less volume reduction than nasal carcinomas. Patients in our study with nasal carcinomas and nasal sarcomas treated with radiation therapy and dorsal rhinotomy had a similar prognosis and survival time overall.

## Limitations

There are limitations of this study. Ideally all patients would have the same number of radiation treatments, same gray for each treatment, and same number of days between completion of radiotherapy and post CT imaging. The small sample size was a major limitation of this study which may reflect a type II statistical error.

In our study, all 15 patients did have a dorsal rhinotomy performed for further cytoreduction at the time of post CT imaging. Additional studies in the future would be needed to evaluate the relation between residual tumor volume and time to recurrent clinical signs without surgery.

In our study both groups had surgical cytoreduction performed following the post CT scan. Evaluation of median survival time compared to volume reduction is difficult to evaluate because of the added variable of surgery. In human medicine multiple studies have shown maximum primary tumor diameter for nasopharyngeal tumors is an independent prognostic factor for overall survival, failure-free survival and local relapse-free survival [20, 21]. Additional studies without surgery and with a larger population are needed to evaluate median survival time relative to volume reduction in canine patients.

## Additional file

**Additional file 1: Figure S1.** Median survival time from starting RT. Survival cure generated by Kaplan–Meier analysis describing survival times of carcinomas and sarcomas from the start of radiation to the time of death.

## Abbreviation

CT: computed tomography
