# Supplementary material for: Evaluation of tumor volume reduction of nasal carcinomas versus sarcomas in dogs treated with definitive fractionated megavoltage radiation: 15 cases (2010–2016)
Source: BMC Res Notes. 2018 Jan 24;11:70. doi: 10.1186/s13104-018-3190-3 (PMC5781340; doi:10.1186/s13104-018-3190-3)
Supplement: Supplementary file 1 [file 13104_2018_3190_MOESM1_ESM.docx]

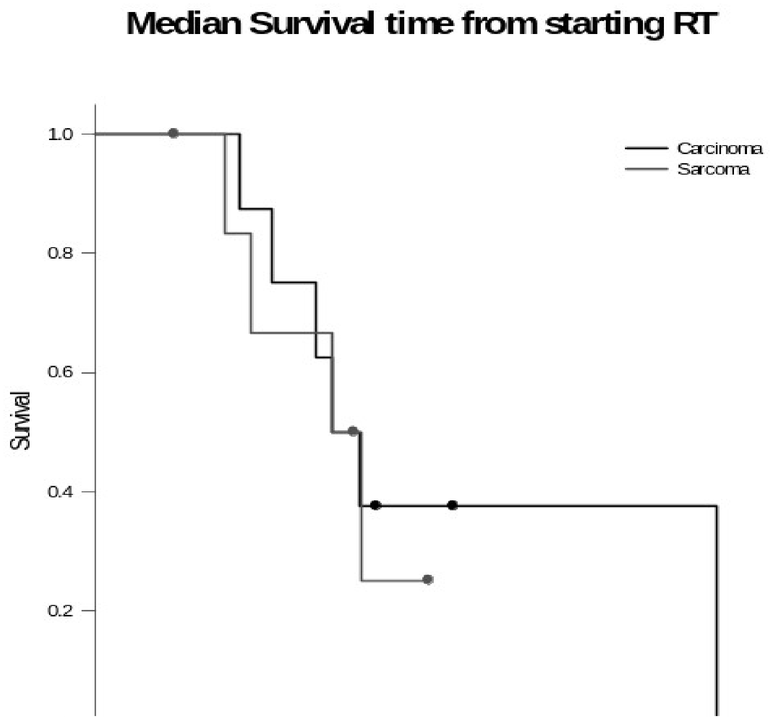

Median Survival time from starting RT
Survival
1.0
0.8
0.6
0.4
0.2
Carcinoma
Sarcoma
